# Induction of neutralizing antibodies in CLL patients after SARS-CoV-2 mRNA vaccination: a monocentric experience

**DOI:** 10.1007/s10238-022-00877-2

**Published:** 2022-09-08

**Authors:** Claudia Baratè, Teresita Caruso, Fabrizio Mavilia, Paola Sammuri, Federico Pratesi, Giuseppe Motta, Valentina Guerri, Sara Galimberti, Paola Migliorini

**Affiliations:** 1 Division of Hematology, Department of Clinical and Experimental Medicine, University of Pisa, Pisa, Italy; 2 Clinical Immunology and Allergy Unit, Department of Clinical and Experimental Medicine, University of Pisa, Via Roma, 67, 56126 Pisa, Italy; 3 General Pathology Unit, Department of Translational Research and New Technologies in Medicine and Surgery, University of Pisa, Pisa, Italy

**Keywords:** Chronic lymphocytic leukemia, SARS-CoV-2 vaccine, Anti-spike antibodies, Neutralizing antibodies, Bruton kinase inhibitors

## Abstract

Vaccination represents the best strategy to fight COVID-19 pandemics, especially in immune compromised subjects. In chronic lymphatic leukemia patients, a marked impairment of the immune response to mRNA SARS-CoV-2 vaccine was observed. In this report, we analyzed anti-RBD and neutralizing antibodies in CLL patients after two doses of mRNA SARS CoV 2 vaccine and evaluated the impact of Bruton kinase inhibitory agents. Twenty-seven CLL patients vaccinated with mRNA vaccines against SARS CoV-2 were recruited. Serum IgG, IgM and IgA anti-RBD antibodies and neutralizing antibodies were detected, and antibody avidity was measured. Peripheral blood leukocytes subsets were evaluated by flow cytometry. After two vaccine doses anti-RBD IgG were produced in 11/27 (40.5%) of patients and levels of IgG and IgA anti RBD in CLL patients were sensibly lower than in controls. Neutralizing antibodies were detectable in 12/27 (44.5%) of the patients and their level was lower than that observed in controls. Disease burden and treatment with Bruton kinases inhibitors markedly impaired vaccine induced antibody response. However, in responder patients, antibody avidity was comparable to normal subjects, indicating that the process of clonal selection and affinity maturation takes place as expected. Taken together, these data confirm the impact of disease burden and therapy on production of anti-RBD and neutralizing antibodies and support the current policy of vaccinating CLL patients.

**Supplementary Information:**

The online version contains supplementary material available at 10.1007/s10238-022-00877-2.

## Introduction

Vaccination represents the best strategy to fight COVID-19 pandemics. Both DNA- and RNA-based vaccines have been approved and nowadays many million people have been vaccinated. Immune compromised subjects were not part of registration vaccine trials, but they obtained a high priority level in the access to vaccination because of their susceptibility to infections.

In chronic lymphocytic leukemia (CLL) patients, either the immune system highly dysregulated by the disease or the immune deficiency exacerbated by anti-leukemic treatment concur in inducing an impairment of immune responses and contribute to increase frequency and severity of infections and to reduce response to vaccines [1]. During treatment, ibrutinib significantly increases CD4+ and CD8+ T cells, especially of effector memory subset, and decreases the Treg/CD4+ T cell ratio. The immunomodulation exerted by ibrutinib is probably due to its off-target action, such as the inhibition of the IL-2 inducible kinase which is mainly expressed by T cells [2]. In acute myeloid leukemia, the BCL2 inhibitor venetoclax enhances T-cell effector function by increasing reactive oxygen species [3]. In CLL, it normalizes B, T, and NK-cell count, reduces the frequency of PD-1+ /CD8+ T cells, but also impairs the NK cells activation, reducing the anti-viral patient’s immunocompetence [4].

Previously, it has been reported that treatment naïve CLL patients respond poorly to HBsAg vaccination [5] or to pneumococcal vaccines, conjugated or not [6], with response rates between 20 and 40%. Immune response to hepatitis vaccine is nearly absent under treatment with BTK inhibitors. Recall responses to zoster vaccine are also reduced by therapy [5].

Several studies evaluated the response of CLL patients to mRNA SARS CoV 2 vaccines, measuring serum anti-spike antibodies after one [7], two [7–9] or three [10] doses. A marked impairment of the immune response was observed, with a response rate of 40–75%. Recently, in a cohort of 286 patients, spike-specific antibody responses were observed in 34% after one and in 75% after two vaccines compared to 94% in healthy donors, especially in cases receiving BTK inhibitors and with low IgA levels [7]. In another series, only 23% of CLL treated patients had detectable antibodies versus 70% of untreated subjects [11]. Among the factors influencing antibody production, in addition to the ongoing therapy also timing of antibody evaluation may account for inter-studies differences.

The spike (S) protein is a complex antigen, and it is conceivable that only part of induced antibodies reacts with the portion of the receptor binding domain (RBD) that interacts with ACE 2 receptor and mediates viral entry into the cells. Thus, evaluation of antibodies that block RBD interaction with ACE 2 represents a better tool to infer protection from COVID-19. So far, only two studies evaluated neutralizing antibodies (NAbs) induced by vaccination in CLL patients; in the first one, the median NAbs inhibition titer was 17% for patients with CLL, Waldenstrom Macroglobulinemia or other non-Hodgkin’s lymphomas versus 32% in controls [12]. In the second study, 160 cancer patients with CLL or other solid tumors exhibited reduced NAbs, especially CLL patients that presented values below the detection limit in 50–60% of the cases [13].

In this report, we analyzed anti-RBD and neutralizing antibodies in CLL patients comparing them with the immune responses observed in healthy individuals after two doses of mRNA SARS CoV 2 vaccine.

## Material and methods

### Patients and methods

Twenty-seven CLL patients regularly followed at the Hematology Unit of Pisa University Hospital and vaccinated with mRNA vaccines (Comirnaty—BNT162b2, *n* = 21; SpikeVax mRNA-1273, *n* = 6) against SARS CoV-2 were recruited into the study.

At the time of enrollment, the following clinical data were collected for each patient: age, gender, stage of disease, treatment status, laboratory parameters and presence of hypogammaglobulinemia (Table 1). Analyses of genomic aberrations by fluorescent in situ hybridization (FISH) and mutational status of the immunoglobulin heavy chain variable (IGHV) gene have been also included.

**Table 1:** CLL patients characteristics at time of serology testing

Age, median, y	70.6
Age < / = 65 y, *N* (%)	
Male sex, *N* (%)	18 (66.7%)
Disease/treatment status, *N* (%)	
Treatment-naive	10 (37%)
On-therapy	13 (48.1%)
Off-therapy in remission	4 (14.9%)
Binet stage (treatment-naive pt) *N* (%)	
A	4 (40%)
B	6 (60%)
C	0 (0%)
IGHV mutational status, *N* (%)	
Mutated	2 (15.4%)
Unmutated	11 (84.6%)
FISH, *N* (%)	
Normal	9 (34.6%)
del(13q)	6 (23.1%)
Trisomy 12	6 (23.1%)
del(11q)	4 (15.4%)
del(17p)	1 (3.8%)
TP53 status	
Wild type	14 (93.3%)
Mutated	1 (6.7%)
Beta2-microglobulin, *N* (%)	
< / = 3.5 mg/L	15 (75%)
> 3.5 mg/L	5 (25%)
Protocols of currently treated, type of target therapy *N* (%)	
BTKis	6 (46.2%)
Venetoclax +− anti-CD20 antibody	4 (30.8%)
others	3 (23%)
Laboratory parameters, median	
ALC (10^9/L)	19.95
Beta2-microglobulin, mg/L	3.18
IgG, mg/dL	767.5
LDH, U/L	212.67
Hb, g/dL	13.72

Twenty-one health care workers (HCW), vaccinated with mRNA BNT162b2, served as control group (mean age ± SD = 46.8 ± 12.9; M/F = 5/16).

Whole blood was collected before the first dose (T0) and 21 days after the second one (T2). Sera were collected and kept frozen at − 60 °C until use.

No patient nor any control previously contracted SARS-CoV-2 infection before recruitment for the study.

The study was approved by the local Ethical Committee (Approval N° 17522) and patients signed an informed consent the day of enrollment.

### FACS analysis of peripheral blood

Peripheral blood granulocytes, monocytes, T and B lymphocytes, NK cells and CD4+ and CD8+ T naïve, central memory (CM) and terminally differentiated effector memory (TEMRA) subsets were evaluated by flow cytometry in 18 out of 27 patients.

The panel used in this study is a mix of anti-CD antibodies (CD4, CD5, CD3, CD19, CD56, CD45, CD8, CD45RA, CCR7, CD27—BD Biosciences, See Table I). We incubated blood samples (100 μl) with the antibody mix for 15 min; erythrolysis was performed with FACS Lysing solution (2 ml, 10 min). Samples were then washed with phosphate buffer saline (PBS) and centrifuged (1000 g, 10 min). Cells were resuspended in PBS and flow cytometric analyses were performed with a BD FACSCanto II flow cytometer. For each sample 100.000 events were analyzed.

### Anti-RBD antibody titers

Antibodies were measured by solid phase assay, on plates coated with recombinant Receptor Binding Domain (SARS-CoV-2 Spike protein aa_319–541_), as previously described [14]. IgG, IgM and IgA anti-RBD antibodies were detected.

### Analysis of neutralizing antibodies

To detect neutralizing antibodies, the kit SPIA (Spike Protein Inhibition Assay, DiaMetra, Perugia, Italy) was employed according to manufacturer’s instructions. In this assay, patient’s antibodies compete with peroxidase-conjugated ACE2 for the binding to viral RBD coated on the solid phase.

Inhibition value was calculated using this formula:

$$\% {\text{ inhibition }} = \, \left[ {{1 } - \, \left( {{\text{Absorbance}}_{{{\text{Sample}}}} } \right)/\left( {{\text{Absorbance}}_{{{\text{Calibrator}}}} } \right)} \right] \, \times {1}00$$

### Avidity assay

Antibody avidity was evaluated in a subgroup of 8 patients, by means of an Avidity ELISA, employing different concentrations of urea as chaotropic reagent. The Avidity Index (AI) was calculated as the extrapolated urea concentration that displaces 50% of serum binding with respect to the control wells using the approach described by Polanec et al. [15]. The area under the curve (AUC) derived by plotting on the y-axis the % binding with respect to the control wells and on x-axis the different Urea molar concentrations was employed to compare the avidity of anti-RBD in vaccinated CLL patients versus vaccinated healthy care workers (HCW).

### Statistical analysis

Statistical analysis was performed using IBM-SPSS® Statistics, and GraphPad Prism statistical packages. Antibody levels at different time points were compared by Kruskall-Wallis. Results of anti-RBD Ig were expressed as Odds Ratio (OR) of a positive internal control set at 1,0. Cut off values have been set at the 97.5th percentile of the healthy care workers (HCW) evaluated before vaccination. *P* < *0.05* was considered as significative.

## Results

Demographic data, disease characteristic and ongoing or previous therapies administered to the 27 enrolled patients are summarized in Table 1. Median age was 70.6 years, with a prevalence of males (66.7%). Binet disease stage was A in 40% and B in 60% of cases. Predominant IgHV mutational status was unmutated (84.6%). Only one patient showed TP53 mutation. About treatment status, 37% of patients were treatment naïve, 48.1% were on therapy and 14.9% were off therapy after achieving clinical remission; 46.2% were on ibrutinib and 30.8% on rituximab plus venetoclax; one patient was on idelalisib.

### Anti-RBD antibodies in vaccinated patients

**Fig. 1:**
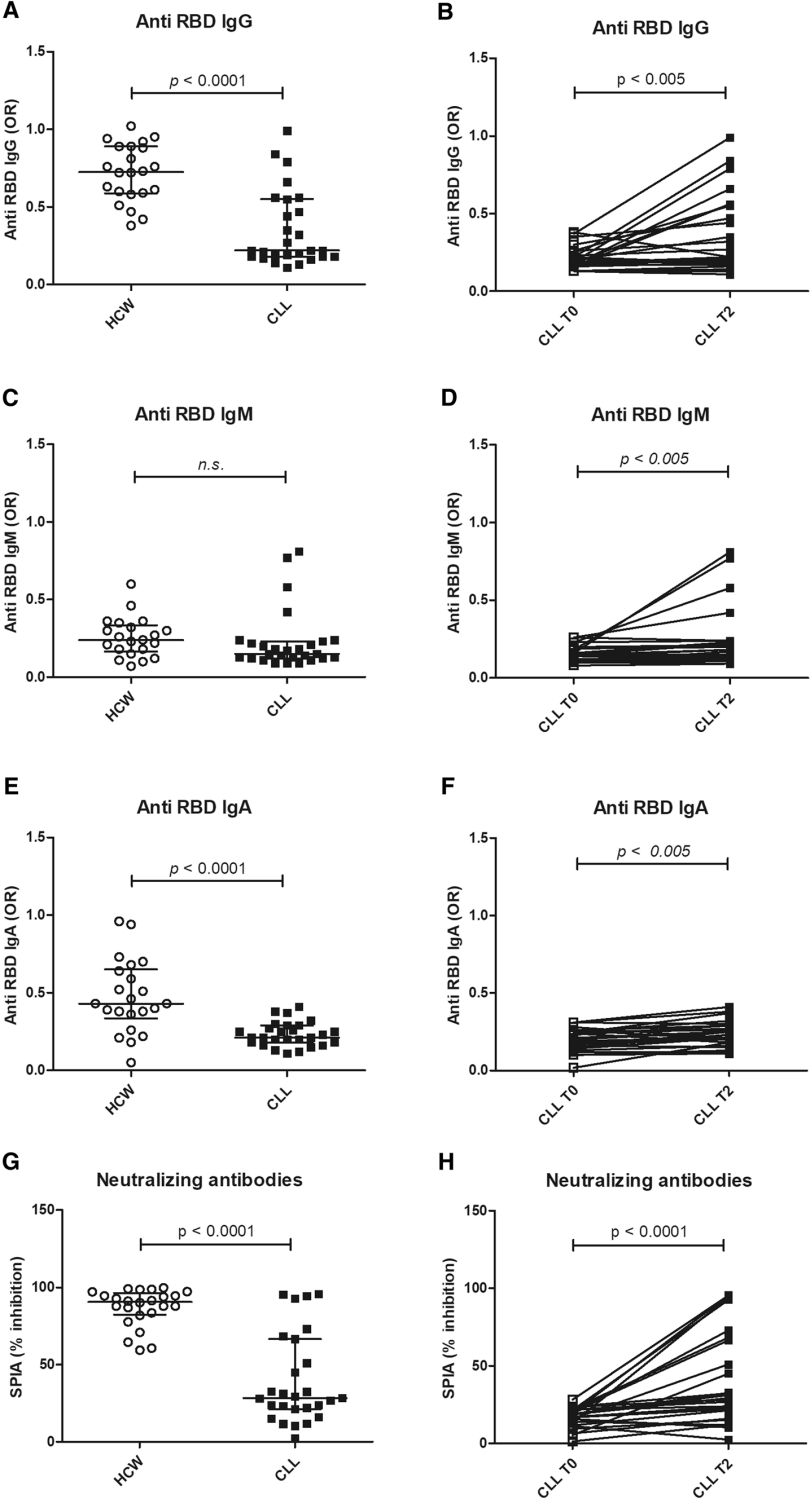
Anti-RBD and neutralizing antibodies in LLC patients. Distribution of IgG (Fig. 1A), IgM (1C) and IgA (1E) anti-RBD and neutralizing antibodies (1G) induced by mRNA vaccine in LLC patients as compared with health care workers (HCW). Levels of IgG (Fig. 1B), IgM (1D) and IgA (1F) anti-RBD and neutralizing antibodies (1H) before the first (T0) and after the second (T2) dose of mRNA vaccine. Results of anti-RBD are represented as odds ratio of a positive internal control (OR). Results of neutralizing antibodies as percentage of inhibition of the binding of ACE to RBD. *p* < 0.05 was considered as significant

After two vaccine doses anti-RBD IgG were produced in 11/27 (40.5%) of patients while IgM and IgA were induced in 4/27 (14.8%) and 5/27 (18.5%), respectively. Levels of IgG and IgA anti RBD in CLL patients were sensibly lower than in HCW used as control (*p* < 0.0001) (Fig. 1A–B, C–D, E–F and Supplementary Figure 1).

As far as the production of NAbs is concerned, antibodies inhibiting the interaction of RBD with ACE2 were detectable in 12/27 (44.5%) of the patients, compared with 100% of healthy controls (*p* < 0.0001), and their level was lower than that observed in controls (*p* < 0.0001). (Fig. 1G–H).

Regarding potential factors influencing vaccine efficacy in CLL patients, we compared the amount of anti-RBD antibodies and NAbs in treated vs untreated patients. Anti-RBD and NAbs were significantly higher in untreated patients (*p* < 0.01 and p < 0.01, respectively).

Conversely, in the subgroup of the 12 patients who developed anti-RBD antibodies after 2 vaccination doses only 3 were under treatment, while 9 were therapy-naïve or far from the last treatment.

**Fig. 2:**
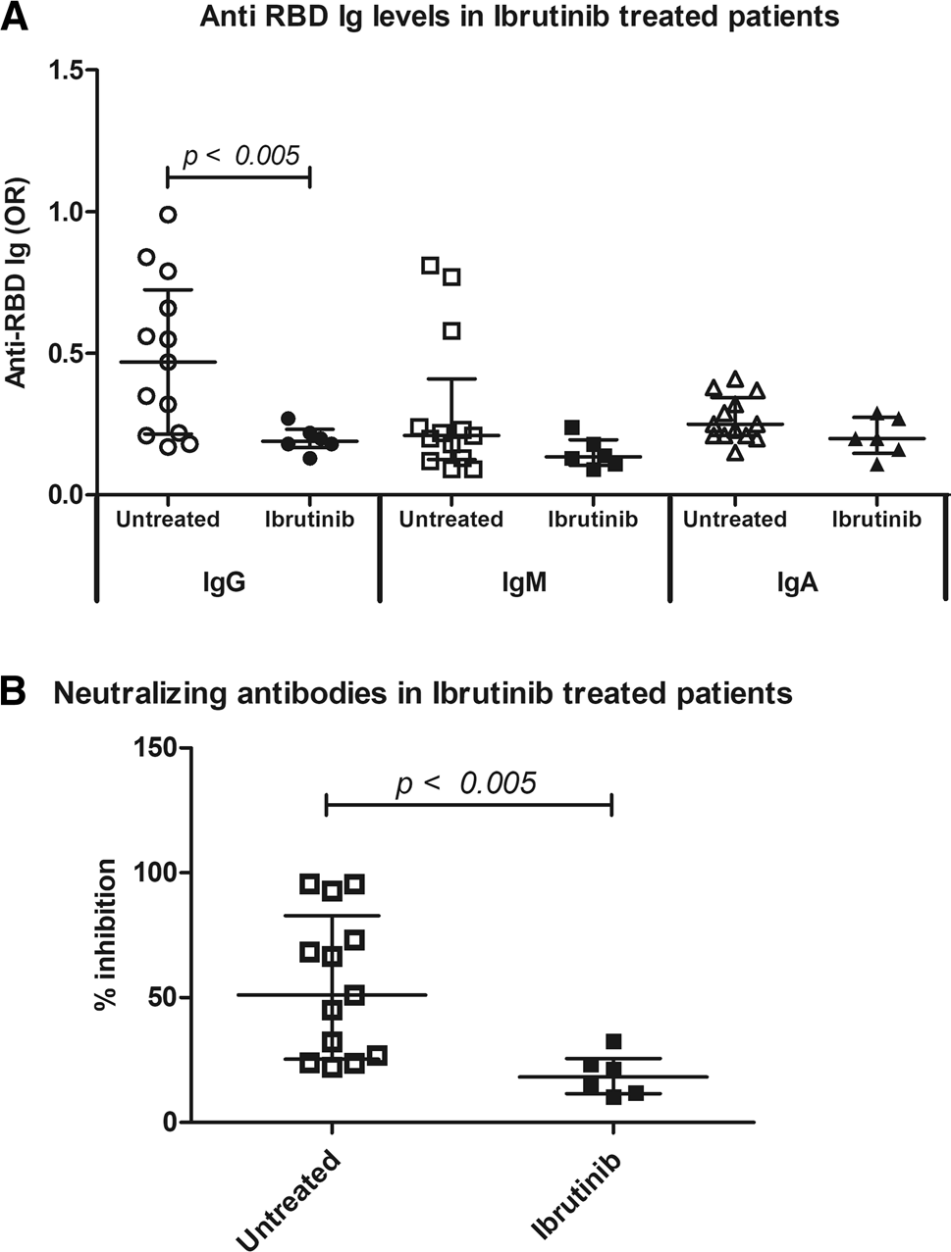
Anti RBD Ab, SPIA and Ibrutinib. Levels of IgG, IgM and IgA anti-RBD (Fig. 2A) and neutralizing antibodies (2B) in LLC patients untreated as compared with patients treated with Ibrutinib. Results of anti-RBD are represented as odds ratio of a positive internal control (OR). Results of neutralizing antibodies as percentage of inhibition of the binding of ACE to RBD. *p* < 0.05 was considered as significant

Among subjects under therapy, only one out of 7 patients (14.3%) receiving ibrutinib produced low amounts of NAbs and the levels of anti-RBD IgG and NAbs were much lower than those measured in controls (*p* < 0.005) (Fig. 2). Out of the 4 patients under venetoclax treatment, only one developed high titers of anti RBD and NAbs.

The titer of Neutr Ab antibodies was inversely correlated with beta-2 microglobulin levels (*p* < 0.005), major lymph node dimension (*p* < 0.005), spleen size (*p* < 0.05) and with affected lymph nodes area (*p* < 0.005) (Supplementary Fig. 2).

The time elapsed between diagnosis and treatment start or between the last therapy and vaccination did not impact on the antibody production.

**Fig. 3:**
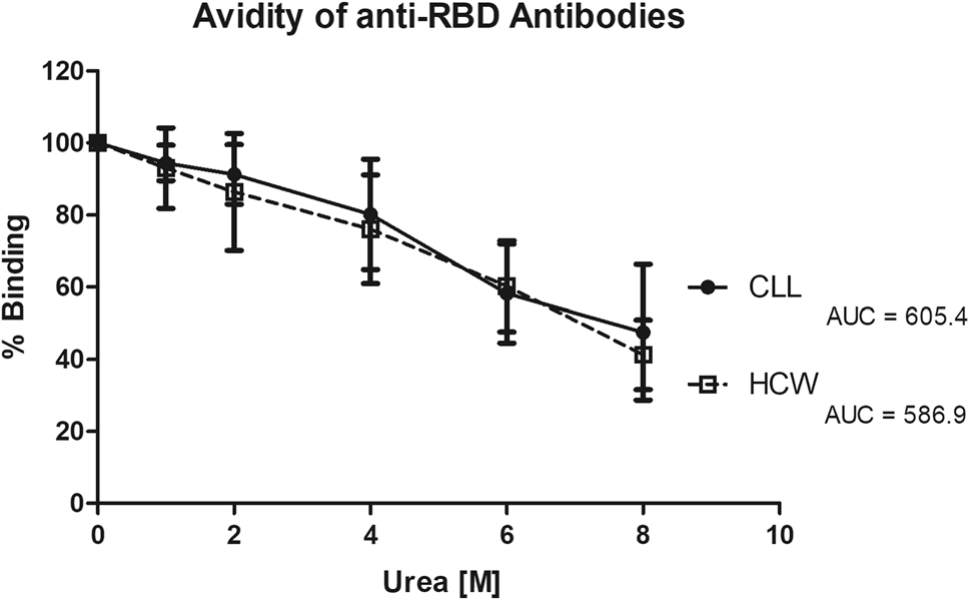
Anti-RBD antibody Avidity in vaccinated LCC and HCW. Antibody avidity was measured by avidity ELISA using different urea concentrations. Curves of binding to RBD obtained with sera from vaccinated CLL patients (●) and with sera from vaccinated health care workers (□) are shown. Results are expressed as the area under the curve (AUC) derived by plotting on the y-axis the % of binding and on x-axis the different Urea molar concentrations

Antibody avidity was evaluated in a subgroup of 8 patients under treatment by means of chaotropic ELISA: mean RBD Avidity Index (AI) was 6.79 ± 1.79 (AUC = 605.9), not statistically different from that obtained for the vaccinated controls (AUC = 586.9) (Fig. 3).

We also analyzed the correlation between vaccine induced anti-RBD or NAbs and peripheral blood cell subset.

We evaluated by flow cytometry the total number of granulocytes, monocytes and lymphocytes (identifying cells by side scatter and CD45 staining); number of T (CD3+), B (CD19+ , CD20+) and NK (CD3−, CD56+) cells; number of CD4+ and CD8+ T naïve (CD27− /CCR7− and CD45RA+), central memory (CD27 + /CCR7 + and CD45RA-) and terminally differentiated effector memory (CD27+ /CCR7+ and CD45RA+) cells.

The levels of NAbs were inversely correlated with baseline WBC number (p < 0.05), total lymphocytes (*p* < 0.05) and B lymphocytes (*p* < 0.05).

On the contrary, CD4+ and CD8+ T memory or naïve cell number did not significantly affect the immune response induced by mRNA vaccination.

## Discussion

Our study, even if conducted in a small cohort, shows that CLL patients poorly respond to SARS CoV2 mRNA vaccines: indeed, anti-RBD antibodies and NAbs are produced only in 40% of vaccinated patients and at lower levels than in vaccinated healthy subjects. These results are comparable to those previously published [8–10, 16]. On the contrary, Parry et al. [7] described low titers of anti-spike antibodies in 75% of the patients after the second dose; clinical features of this cohort, and namely the higher proportion of treatment-naïve patients, may explain this discordance.

The production of NAbs has also been evaluated in other studies that report positive results in 40 to 50% of the patients [12] [13, 17], analogously to our series.

NAbs have been evaluated by means of an assay based on the inhibition of RBD-ACE2 interaction. Plaque reduction neutralization tests represent the golden standard for the detection of neutralizing antibodies. However, a positive correlation between neutralization assays and inhibition of RBD-ACE2 interaction has been obtained by many authors and the antibody-mediated blockage of ACE2-spike interaction has been considered a SARS-CoV-2 surrogate virus neutralization test [18].

Interestingly, notwithstanding the low percentage of serological immune response, we demonstrated that in responder patients, antibody avidity was comparable to normal subjects, indicating that the process of clonal selection and affinity maturation takes place as expected. This observation is relevant, because it sustains once again the need of proceeding with vaccination also in CLL patients.

The possibility that the use of different vaccines may partially improve the immune response of CLL patients cannot be completely ruled out. A lower titer of antibodies has been detected in patients vaccinated with BNT162b2 vs mRNA-1273 [16]. In our study, most patients were vaccinated with BNT162b2 only a few patients were vaccinated with mRNA-1273 vaccine and their antibody titer was not different.

As shown in the present study, disease burden, ongoing and past therapies are the main factors affecting antibody responses. As previously reported, in the present report we observed lower antibody levels in patients treated with ibrutinib; because of the low number of patients receiving other treatments, data on venetoclax or chlorambucil need further investigation.

BTK inhibitors, irreversibly inactivating BTK, interfere with BCR signaling and thus with B cell development, proliferation, differentiation and activation. Thus, it is conceivable that a therapy directly targeting B cells may impair antibody responses to novel antigens. However, reducing the number of exhausted T cells and immunosuppressive Treg, BTK inhibitors may restore a normal T cell compartment and also favor dendritic cell maturation, thus positively affecting immune responses [19].

In addition to B cells, also T cell response is essential to protect against Coronavirus: specific IFN-y and IL-2-mediated immune responses were observed in CLL patients irrespective of anti-S production or neutralizing activity [20]. Even if we did not evaluate anti-SARS CoV 2 T responses, the phenotype of peripheral T cells in our patients is compatible with a normal T response. Cellular immunity to spike should be further evaluated in CLL vaccinated subjects to get more insights on the level of protection that can be achieved by vaccination.

Herishanu [21] recently reported a case series of persistence of SARS-CoV-2 antibodies after the second BNT162b2 mRNA COVID-19 vaccine dose in CLL patients. At 6 months after the second vaccine dose antibody titers were lower in patients compared with controls, but still detectable, and patients on active treatment have the lowest titers.

In a series of 536 Italian patients with different hematological malignancies 37% died; if compared with chronic myeloid malignancies, the probability of death for CLL patients was 60% higher [22]. In another CLL cohort, where 79% of the patients had been hospitalized for COVID-19, CLL disease burden significantly impacted on outcome: milder disease was observed in untreated patients, age did not impact on mortality and BTK inhibitors appeared to exert a protective effect, as observed in the CLL patients we studied [23].

A restoration of cellular and humoral immune function can be induced also by target therapy and this ability, coupled with the anti-inflammatory action played by some inhibitors such as ibrutinib, explains why continuing these therapies during moderate COVID-19 infection could be useful.

The recent approval by EMA of tixagevimab and cilgavimab (website: https://www.ema.europa.eu/en/medicines/human/EPAR/evusheld) offers new opportunities to non-responder patients. These antibodies have been designed to attach to the spike protein at two different sites, avoiding the virus to enter the cells, to multiply and cause COVID-19. The combination drug is indicated for prophylaxis of COVID-19 in adults and adolescents with active hematological malignancies, those who received allogeneic transplantation, in active treatment with high-dose corticosteroids, alkylating agents, antimetabolites, transplant-related immunosuppressive drugs, cancer chemotherapeutic agents, including B-cell–depleting agents in CLL.

Taken together, all available data support the current policy of vaccinating CLL patients, irrespective of the ongoing therapy and suggest that measurement of anti-RBD and neutralizing antibodies might help in planning passive immunotherapy with anti-Spike antibodies.

## Supplementary Information

Below is the link to the electronic supplementary material.

Supplementary file1 (TIF 2024 kb)

Supplementary file2 (TIF 1705 kb)

Supplementary file3 (DOCX 12 kb)
