# Supplementary figures and images for: Induction of neutralizing antibodies in CLL patients after SARS-CoV-2 mRNA vaccination: a monocentric experience
Source: Clin Exp Med. 2022 Sep 8;23(4):1197–203. doi: 10.1007/s10238-022-00877-2 (PMC9453722; doi:10.1007/s10238-022-00877-2)

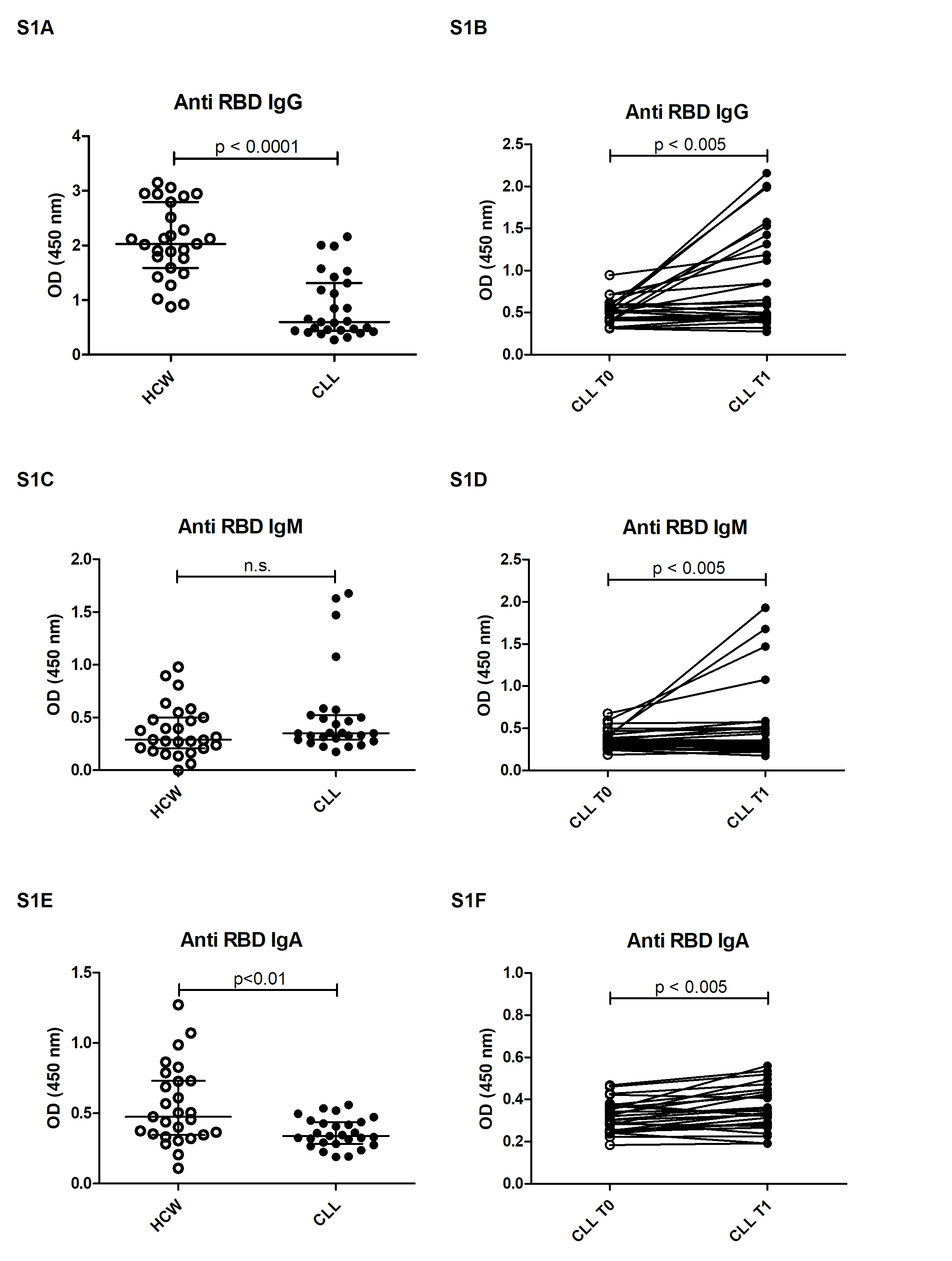

Supplement: Supplementary file 1 — Supplementary file1 (TIF 2024 kb) [file 10238_2022_877_MOESM1_ESM.tif]

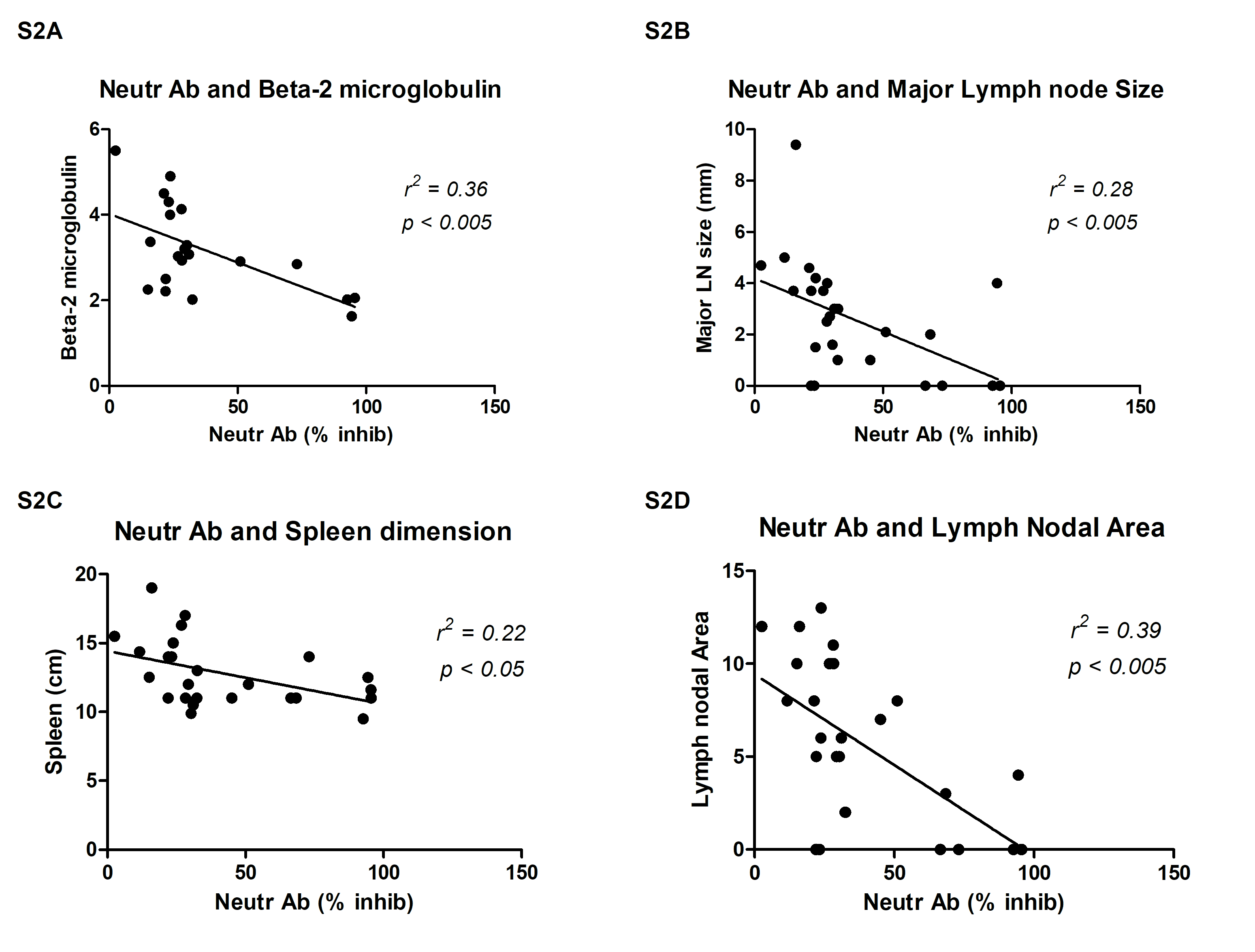

Supplement: Supplementary file 2 — Supplementary file2 (TIF 1705 kb) [file 10238_2022_877_MOESM2_ESM.tif]
