# Supplementary material for: Induction of neutralizing antibodies in CLL patients after SARS-CoV-2 mRNA vaccination: a monocentric experience
Source: Clin Exp Med. 2022 Sep 8;23(4):1197–203. doi: 10.1007/s10238-022-00877-2 (PMC9453722; doi:10.1007/s10238-022-00877-2)
Supplement: Supplementary file 3 — Supplementary file3 (DOCX 12 kb) [file 10238_2022_877_MOESM3_ESM.docx]

**Suppl. Fig 1: Anti-RBD antibodies in LLC patients**

Distribution of IgG (Fig. S1A), IgM (S1C) and IgA (S1E) anti-RBD induced by mRNA vaccine in LLC patients as compared with health care workers (HCW).

Levels of IgG (Fig. S1B), IgM (S1D) and IgA (S1F) anti-RBD before the first (T0) and after the second (T2) dose of mRNA vaccine.

Results of anti-RBD are represented as optical density at 450 nm (OD450). *p* < 0.05 was considered as significant.

**Suppl. Fig 2. Correlation of Neutralizing antibodies and clinical parameters in vaccinated LCC patients**

Neutralizing antibodies were measured by the Spike Protein Inhibition Assay and expressed as percentage of inhibition of the interaction of RBD with ACE2.

Neutralizing antibodies correlated with Beta 2 microglobulin levels (3A), major lymph node size (3B), spleen dimension (3C) and lymph nodal area (3D). *p* < 0.05 was considered as significant.
